# 5*H*-Thio­chromeno[2,3-*b*]pyridine-5,10,10-trione

**DOI:** 10.1107/S1600536810038171

**Published:** 2010-09-30

**Authors:** Muhammad Naeem Khan, M. Nawaz Tahir, Misbahul Ain Khan, Salma Rehman, Abdul Qayyum Ather

**Affiliations:** a Department of Chemistry, Islamia University, Bahawalpur, Pakistan; b Applied Chemistry Research Center, PCSIR Laboratories Complex, Lahore 54600, Pakistan; c Department of Physics, University of Sargodha, Sargodha, Pakistan

## Abstract

The asymmetric unit of the title compound, C_12_H_7_NO_3_S, contains two independent mol­ecules with different geometric­al configurations. The dihedral angles between the benzene and pyridine rings in the two mol­ecules are 3.7 (2) and 5.40 (19)°. The central heterocyclic fused rings have different puckering parameters [*Q* = 0.122 (3) Å, θ = 100.4 (13), ϕ = 185.3 (19)° in one mol­ecule, 0.101 (3) Å, 101.4 (3) and 2 (2)° in the other]. The SO_2_ group is oriented at dihedral angles of 81.06 (14) and 82.58 (15)° with the benzene and pyridine rings, respectively, in one mol­ecule [87.21 (14) and 87.66 (14)° in the second]. In the crystal, the mol­ecules are linked into zigzag polymeric chains along the *b* axis by inter­molecular C—H⋯O hydrogen bonding. π–π inter­actions with centroid–centroid distances in the range 3.825 (3)–4.153 (3) Å stabilize the structure. S—O⋯π and C—O⋯π inter­actions are also observed.

## Related literature

For background to our work on pyridine- and thio-containing heterocyclic rings and for related structures, see: Khan *et al.* (2008**a* ▶,b* ▶). For the preparation, see: Khan *et al.* (2008**a* ▶,b* ▶); Kruger & Mann (1954 ▶). For puckering parameters, see: Cremer & Pople (1975 ▶).
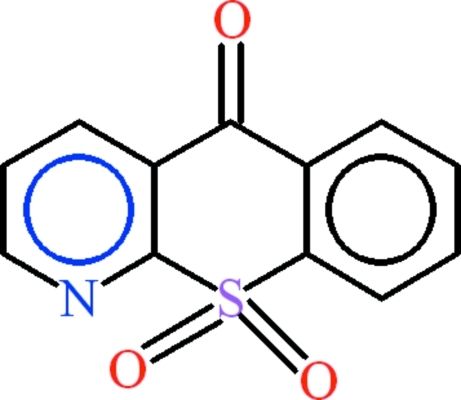


## Experimental

### 

#### Crystal data

C_12_H_7_NO_3_S
*M* *_r_* = 245.25
Orthorhombic, 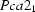

*a* = 12.157 (5) Å
*b* = 11.483 (5) Å
*c* = 14.964 (7) Å
*V* = 2089.0 (16) Å^3^
*Z* = 8
Mo *K*α radiation
μ = 0.30 mm^−1^
*T* = 296 K
0.35 × 0.14 × 0.12 mm

#### Data collection

Bruker Kappa APEXII CCD diffractometer
Absorption correction: multi-scan (*SADABS*; Bruker, 2005 ▶) *T*_min_ = 0.968, *T*_max_ = 0.985
29502 measured reflections
3718 independent reflections
2838 reflections with *I* > 2σ(*I*)
*R*_int_ = 0.061

#### Refinement

*R*[*F* ^2^ > 2σ(*F* ^2^)] = 0.041
*wR*(*F* ^2^) = 0.094
*S* = 1.04
3718 reflections
307 parameters
1 restraint
H-atom parameters constrained
Δρ_max_ = 0.20 e Å^−3^
Δρ_min_ = −0.25 e Å^−3^
Absolute structure: Flack (1983 ▶), 1749 Friedel pairs
Flack parameter: 0.13 (9)

### 

Data collection: *APEX2* (Bruker, 2009 ▶); cell refinement: *SAINT* (Bruker, 2009 ▶); data reduction: *SAINT*; program(s) used to solve structure: *SHELXS97* (Sheldrick, 2008 ▶); program(s) used to refine structure: *SHELXL97* (Sheldrick, 2008 ▶); molecular graphics: *ORTEP-3 for Windows* (Farrugia, 1997 ▶) and *PLATON* (Spek, 2009 ▶); software used to prepare material for publication: *WinGX* (Farrugia, 1999 ▶) and *PLATON*.

## Supplementary Material

Crystal structure: contains datablocks global, I. DOI: 10.1107/S1600536810038171/bq2235sup1.cif

Structure factors: contains datablocks I. DOI: 10.1107/S1600536810038171/bq2235Isup2.hkl

Additional supplementary materials:  crystallographic information; 3D view; checkCIF report

## Figures and Tables

**Table 1:** Hydrogen-bond geometry (Å, °) *Cg*1 and *Cg*2 are the centroids of the S1/C1/C6/C7/C8/C12 and S2/C13/C18/C19/C20/C24 rings, respectively.

*D*—H⋯*A*	*D*—H	H⋯*A*	*D*⋯*A*	*D*—H⋯*A*
C3—H3⋯O5^i^	0.93	2.59	3.372 (5)	142
C4—H4⋯O3^ii^	0.93	2.57	3.472 (5)	164
C15—H15⋯O2^iii^	0.93	2.58	3.367 (5)	143
S1—O3⋯*Cg*2^iii^	1.43 (1)	3.21 (1)	4.421 (3)	141 (1)
C19—O4⋯*Cg*1^ii^	1.21 (1)	2.87 (1)	3.585 (4)	117 (1)

Symmetry codes: (i) 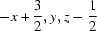; (ii) 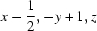; (iii) 
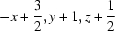
.

**Fig. 1.**
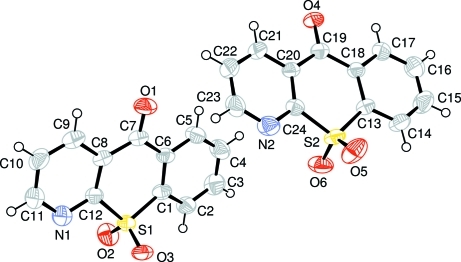
View of the title compound with the atom numbering scheme. The thermal displacements are drawn at the 50% probability level.

**Fig. 2.**
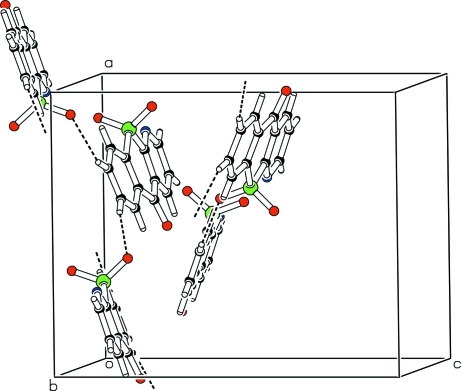
The partial packing (PLATON; Spek, 2009) which shows that molecules form polymeric chains extending along the b axis.

## supplementary crystallographic information

### Comment

The title compound (I, Fig. 1) is an extension to our work related to pyridine and thio containing heterocyclic rings (Khan *et al.*, 2008**a*, b*). We have reported previously the crystal structures of (II) *i.e.* 7-nitro-5*H*-1-benzothiopyrano[2,3-*b*]-pyridin-5-one (Khan *et al.*, 2008*a*) and 5*H*-1-benzothiopyrano[2,3-*b*]pyridin-5-one (Khan *et al.*, 2008*b*), which are related to the title compound.

The title compound consist of two independent molecules having different configuration. In one molecule, the phenyl ring A (C1—C6) and pyridine ring B (C8—C11/N1/C12) are planar with r. m. s. deviation of 0.0077 and 0.0063 Å, respectively. The dihedral angle between A/B is 5.40 (19)°. The heterocyclic central fused ring C (S1/C1/C6—C8/C12) is slightly twisted with puckering parameters (Cremer & Pople, 1975) given by Q = 0.122 (3) Å, θ = 100.4 (14)°, φ = 185.3 (18)°. The SO_2_ group D (O2/S1/O3) of this molecule makes dihedral angle of 87.21 (14) and 87.66 (14) ° with the phenyl ring A and pyridine ring B, respectively. In the second molecule, the phenyl ring E (C13—C18) and pyridine ring F (C20—C23/N2/C24) are planar with r. m. s. deviation of 0.0040 and 0.0018 Å, respectively. The dihedral angle between E/F is 3.72 (20)°. The puckering parameters of the central fused ring G (S2/C13/C18—C20/C24) are given by Q = 0.101 (3) Å, θ = 101.4 (3)°, φ = 2(2)°. In this molecule, the SO_2_ group H (O5/S2/O6) makes dihedral angle of 81.06 (14) and 82.58 (15)° with the parent phenyl ring E and pyridine ring F, respectively. There exist intermolecular H-bonding of C—H···O type (Table 1) due to which molecules establish zigzag polymeric chains. The π–π interactions in the range of 3.825 (3)–4.153 (3) Å exist which plays important role in stabilizing the molecules.

### Experimental

5*H*-1-Benzothiopyrano[2,3-*b*]pyridin-5-one was prepared freshly (Khan *et al.*, 2008*b*) and was oxidized using acetic acid and hydrogen peroxide according to the method described by Kruger & Mann, 1954.

### Refinement

The H-atoms were positioned geometrically (C–H = 0.93 Å) and refined as riding with *U*_iso_(H) = x*U*_eq_(C), where x = 1.2 for all aryl H-atoms.

### Crystal data

**Table:**

C_12_H_7_NO_3_S	*F*(000) = 1008
*M**_r_* = 245.25	*D*_x_ = 1.560 Mg m^−^^3^
Orthorhombic, *P**c**a*2_1_	Mo *K*α radiation, λ = 0.71073 Å
Hall symbol: P 2c -2ac	Cell parameters from 2838 reflections
*a* = 12.157 (5) Å	θ = 2.2–25.2°
*b* = 11.483 (5) Å	µ = 0.30 mm^−^^1^
*c* = 14.964 (7) Å	*T* = 296 K
*V* = 2089.0 (16) Å^3^	Needle, white
*Z* = 8	0.35 × 0.14 × 0.12 mm

### Data collection

**Table:**

Bruker Kappa APEXII CCD diffractometer	3718 independent reflections
Radiation source: fine-focus sealed tube	2838 reflections with *I* > 2σ(*I*)
graphite	*R*_int_ = 0.061
Detector resolution: 8.10 pixels mm^-1^	θ_max_ = 25.2°, θ_min_ = 2.4°
ω scans	*h* = −14→14
Absorption correction: multi-scan (*SADABS*; Bruker, 2005)	*k* = −13→13
*T*_min_ = 0.968, *T*_max_ = 0.985	*l* = −17→17
29502 measured reflections	

### Refinement

**Table:**

Refinement on *F*^2^	Secondary atom site location: difference Fourier map
Least-squares matrix: full	Hydrogen site location: inferred from neighbouring sites
*R*[*F*^2^ > 2σ(*F*^2^)] = 0.041	H-atom parameters constrained
*wR*(*F*^2^) = 0.094	*w* = 1/[σ^2^(*F*_o_^2^) + (0.043*P*)^2^ + 0.3395*P*] where *P* = (*F*_o_^2^ + 2*F*_c_^2^)/3
*S* = 1.04	(Δ/σ)_max_ < 0.001
3718 reflections	Δρ_max_ = 0.20 e Å^−^^3^
307 parameters	Δρ_min_ = −0.25 e Å^−^^3^
1 restraint	Absolute structure: Flack (1983), 1749 Friedal pairs
Primary atom site location: structure-invariant direct methods	Flack parameter: 0.13 (9)

### Special details

**Table:**

Geometry. Bond distances, angles *etc*. have been calculated using the rounded fractional coordinates. All su's are estimated from the variances of the (full) variance-covariance matrix. The cell e.s.d.'s are taken into account in the estimation of distances, angles and torsion angles
Refinement. Refinement of *F*^2^ against ALL reflections. The weighted *R*-factor *wR* and goodness of fit *S* are based on *F*^2^, conventional *R*-factors *R* are based on *F*, with *F* set to zero for negative *F*^2^. The threshold expression of *F*^2^ > σ(*F*^2^) is used only for calculating *R*-factors(gt) *etc*. and is not relevant to the choice of reflections for refinement. *R*-factors based on *F*^2^ are statistically about twice as large as those based on *F*, and *R*- factors based on ALL data will be even larger.

### Fractional atomic coordinates and isotropic or equivalent isotropic displacement parameters (Å^2^)

**Table:**

	*x*	*y*	*z*	*U*_iso_*/*U*_eq_	
S1	0.81953 (6)	0.23202 (7)	0.11098 (7)	0.0396 (3)	
O1	0.4807 (2)	0.2221 (2)	0.2163 (2)	0.0604 (10)	
O2	0.8585 (2)	0.2231 (2)	0.0215 (2)	0.0605 (10)	
O3	0.90038 (19)	0.2461 (2)	0.1799 (2)	0.0515 (10)	
N1	0.7975 (3)	0.0089 (3)	0.1253 (4)	0.0579 (16)	
C1	0.7263 (2)	0.3484 (3)	0.1177 (2)	0.0396 (11)	
C2	0.7670 (3)	0.4540 (3)	0.0899 (3)	0.0480 (14)	
C3	0.7021 (3)	0.5509 (4)	0.0970 (3)	0.0620 (17)	
C4	0.5960 (3)	0.5429 (4)	0.1313 (3)	0.0603 (18)	
C5	0.5556 (3)	0.4373 (4)	0.1565 (3)	0.0517 (16)	
C6	0.6192 (3)	0.3367 (3)	0.1509 (2)	0.0394 (11)	
C7	0.5707 (3)	0.2240 (3)	0.1802 (2)	0.0410 (12)	
C8	0.6319 (3)	0.1143 (3)	0.1664 (3)	0.0407 (11)	
C9	0.5807 (3)	0.0072 (3)	0.1860 (4)	0.0523 (18)	
C10	0.6387 (4)	−0.0949 (3)	0.1760 (3)	0.0590 (19)	
C11	0.7462 (4)	−0.0915 (4)	0.1467 (3)	0.0620 (18)	
C12	0.7393 (3)	0.1068 (3)	0.1364 (3)	0.0389 (14)	
S2	0.55676 (6)	0.72967 (8)	0.42422 (7)	0.0439 (3)	
O4	0.2126 (2)	0.7236 (2)	0.33032 (18)	0.0491 (9)	
O5	0.5968 (2)	0.7223 (2)	0.5146 (2)	0.0701 (11)	
O6	0.6358 (2)	0.7403 (2)	0.3539 (2)	0.0635 (10)	
N2	0.5313 (3)	0.5063 (3)	0.4106 (3)	0.0539 (13)	
C13	0.4655 (2)	0.8471 (3)	0.4173 (3)	0.0391 (11)	
C14	0.5086 (3)	0.9534 (3)	0.4424 (3)	0.0550 (16)	
C15	0.4448 (3)	1.0509 (4)	0.4341 (4)	0.0687 (19)	
C16	0.3396 (3)	1.0443 (4)	0.4026 (4)	0.0660 (19)	
C17	0.2959 (3)	0.9386 (3)	0.3784 (3)	0.0533 (16)	
C18	0.3576 (3)	0.8374 (3)	0.3856 (2)	0.0392 (11)	
C19	0.3053 (3)	0.7249 (3)	0.3592 (2)	0.0392 (11)	
C20	0.3670 (3)	0.6127 (3)	0.3704 (3)	0.0365 (11)	
C21	0.3156 (3)	0.5088 (3)	0.3504 (4)	0.0473 (16)	
C22	0.3716 (3)	0.4060 (3)	0.3600 (3)	0.0540 (16)	
C23	0.4783 (4)	0.4074 (4)	0.3899 (3)	0.0567 (19)	
C24	0.4750 (3)	0.6059 (3)	0.4004 (3)	0.0384 (11)	
H2	0.83773	0.45951	0.06657	0.0578*	
H3	0.72920	0.62284	0.07869	0.0744*	
H4	0.55289	0.60938	0.13709	0.0725*	
H5	0.48391	0.43225	0.17780	0.0621*	
H9	0.50812	0.00552	0.20555	0.0627*	
H10	0.60550	−0.16592	0.18901	0.0705*	
H11	0.78485	−0.16105	0.14140	0.0741*	
H14	0.57990	0.95872	0.46456	0.0658*	
H15	0.47355	1.12296	0.45013	0.0823*	
H16	0.29745	1.11160	0.39751	0.0790*	
H17	0.22412	0.93488	0.35701	0.0641*	
H21	0.24310	0.50852	0.33052	0.0567*	
H22	0.33746	0.33569	0.34637	0.0648*	
H23	0.51535	0.33705	0.39606	0.0682*	

### Atomic displacement parameters (Å^2^)

**Table:**

	*U*^11^	*U*^22^	*U*^33^	*U*^12^	*U*^13^	*U*^23^
S1	0.0312 (4)	0.0449 (5)	0.0428 (5)	−0.0024 (4)	0.0075 (4)	0.0005 (6)
O1	0.0356 (14)	0.0615 (19)	0.084 (2)	−0.0044 (13)	0.0160 (15)	0.0022 (16)
O2	0.0675 (17)	0.063 (2)	0.0510 (16)	−0.0010 (15)	0.0260 (15)	0.0026 (15)
O3	0.0278 (12)	0.0550 (18)	0.0716 (19)	−0.0022 (12)	−0.0036 (12)	0.0009 (14)
N1	0.0506 (19)	0.046 (2)	0.077 (4)	−0.0001 (16)	0.001 (2)	−0.0009 (19)
C1	0.0339 (18)	0.048 (2)	0.037 (2)	−0.0020 (15)	−0.0044 (17)	0.0073 (19)
C2	0.041 (2)	0.044 (2)	0.059 (3)	−0.0074 (18)	0.012 (2)	0.008 (2)
C3	0.066 (3)	0.047 (3)	0.073 (3)	−0.004 (2)	−0.002 (3)	0.013 (2)
C4	0.056 (2)	0.046 (3)	0.079 (4)	0.013 (2)	0.001 (2)	0.007 (2)
C5	0.039 (2)	0.054 (3)	0.062 (3)	0.007 (2)	0.002 (2)	0.004 (2)
C6	0.0312 (18)	0.048 (2)	0.039 (2)	0.0023 (16)	−0.0043 (15)	0.0007 (16)
C7	0.031 (2)	0.049 (2)	0.043 (2)	−0.0046 (17)	−0.0005 (16)	0.0049 (18)
C8	0.0320 (19)	0.047 (2)	0.043 (2)	−0.0072 (18)	−0.0023 (17)	0.0030 (19)
C9	0.041 (2)	0.060 (3)	0.056 (4)	−0.011 (2)	−0.002 (2)	0.007 (2)
C10	0.058 (3)	0.042 (3)	0.077 (4)	−0.012 (2)	−0.006 (2)	0.006 (2)
C11	0.059 (2)	0.043 (3)	0.084 (4)	−0.002 (2)	0.002 (3)	−0.004 (2)
C12	0.0348 (19)	0.041 (2)	0.041 (3)	0.0000 (17)	−0.0007 (17)	0.0009 (18)
S2	0.0301 (4)	0.0465 (5)	0.0551 (6)	−0.0046 (4)	−0.0106 (4)	0.0082 (7)
O4	0.0295 (14)	0.0615 (17)	0.0564 (18)	0.0004 (12)	−0.0134 (13)	−0.0057 (14)
O5	0.0752 (19)	0.065 (2)	0.070 (2)	−0.0092 (15)	−0.0416 (18)	0.0119 (16)
O6	0.0373 (15)	0.0631 (19)	0.090 (2)	−0.0025 (13)	0.0133 (15)	0.0150 (15)
N2	0.0447 (17)	0.049 (2)	0.068 (3)	0.0048 (15)	−0.007 (2)	0.0099 (17)
C13	0.0334 (18)	0.043 (2)	0.041 (2)	−0.0044 (15)	−0.0053 (17)	0.0032 (18)
C14	0.041 (2)	0.052 (3)	0.072 (3)	−0.009 (2)	−0.011 (2)	0.003 (2)
C15	0.070 (3)	0.044 (3)	0.092 (4)	−0.009 (2)	−0.012 (3)	−0.004 (3)
C16	0.062 (3)	0.048 (3)	0.088 (4)	0.012 (2)	−0.004 (3)	−0.005 (3)
C17	0.042 (2)	0.053 (3)	0.065 (3)	0.0102 (19)	−0.007 (2)	−0.010 (2)
C18	0.0316 (18)	0.047 (2)	0.039 (2)	0.0004 (16)	−0.0020 (15)	−0.0030 (15)
C19	0.034 (2)	0.052 (2)	0.0317 (19)	−0.0013 (17)	0.0002 (16)	0.0005 (17)
C20	0.0294 (18)	0.044 (2)	0.036 (2)	−0.0040 (17)	0.0018 (15)	0.0006 (17)
C21	0.039 (2)	0.049 (3)	0.054 (3)	−0.0090 (19)	0.002 (2)	−0.004 (2)
C22	0.051 (2)	0.044 (3)	0.067 (3)	−0.012 (2)	0.009 (2)	−0.004 (2)
C23	0.058 (3)	0.037 (3)	0.075 (4)	0.008 (2)	0.006 (2)	0.003 (2)
C24	0.0362 (19)	0.039 (2)	0.040 (2)	0.0010 (17)	0.0031 (17)	0.0052 (18)

### Geometric parameters (Å, °)

**Table:**

S1—O2	1.424 (3)	C2—H2	0.9300
S1—O3	1.434 (3)	C3—H3	0.9300
S1—C1	1.755 (3)	C4—H4	0.9300
S1—C12	1.779 (4)	C5—H5	0.9300
S2—O5	1.440 (3)	C9—H9	0.9300
S2—O6	1.430 (3)	C10—H10	0.9300
S2—C13	1.749 (3)	C11—H11	0.9300
S2—C24	1.771 (4)	C13—C14	1.380 (5)
O1—C7	1.220 (4)	C13—C18	1.399 (5)
O4—C19	1.207 (4)	C14—C15	1.368 (6)
N1—C11	1.349 (6)	C15—C16	1.365 (6)
N1—C12	1.339 (5)	C16—C17	1.374 (6)
N2—C23	1.342 (6)	C17—C18	1.387 (5)
N2—C24	1.342 (5)	C18—C19	1.493 (5)
C1—C2	1.374 (5)	C19—C20	1.500 (5)
C1—C6	1.400 (4)	C20—C21	1.380 (5)
C2—C3	1.368 (6)	C20—C24	1.390 (5)
C3—C4	1.391 (5)	C21—C22	1.370 (5)
C4—C5	1.362 (6)	C22—C23	1.372 (6)
C5—C6	1.393 (6)	C14—H14	0.9300
C6—C7	1.488 (5)	C15—H15	0.9300
C7—C8	1.478 (5)	C16—H16	0.9300
C8—C12	1.383 (5)	C17—H17	0.9300
C8—C9	1.409 (5)	C21—H21	0.9300
C9—C10	1.376 (5)	C22—H22	0.9300
C10—C11	1.379 (7)	C23—H23	0.9300
			
O1···C22	3.293 (5)	N2···O6	3.091 (5)
O1···C23	3.358 (6)	N2···O5	3.035 (5)
O1···O5^i^	3.226 (4)	N1···H14^iii^	2.8900
O1···C11^ii^	3.385 (6)	N1···H9^iv^	2.8300
O2···C17^i^	3.400 (5)	N2···H2^viii^	2.8800
O2···N1	3.002 (5)	N2···H21^v^	2.8500
O2···O4^i^	3.051 (4)	C1···O4^v^	3.292 (4)
O2···C15^iii^	3.367 (5)	C3···O5^x^	3.372 (5)
O2···C18^i^	3.394 (5)	C5···O5^i^	3.362 (5)
O2···C19^i^	3.197 (5)	C6···O4^v^	2.996 (4)
O3···N1	3.107 (5)	C6···O5^i^	3.393 (5)
O3···C10^iv^	3.378 (5)	C7···O4^v^	2.896 (4)
O3···C19^v^	2.940 (4)	C7···O5^i^	3.266 (5)
O3···O4^v^	3.225 (4)	C8···O4^v^	3.232 (5)
O3···C20^v^	3.305 (5)	C10···O6^xiii^	3.266 (5)
O3···C18^v^	3.266 (4)	C10···O3^ii^	3.378 (5)
O4···O3^vi^	3.225 (4)	C11···O1^iv^	3.385 (6)
O4···O2^vii^	3.051 (4)	C15···O2^xi^	3.367 (5)
O4···C23^vi^	3.342 (6)	C17···O2^vii^	3.400 (5)
O4···C6^vi^	2.996 (4)	C18···O3^vi^	3.266 (4)
O4···C1^vi^	3.292 (4)	C18···O2^vii^	3.394 (5)
O4···C8^vi^	3.232 (5)	C19···O2^vii^	3.197 (5)
O4···C7^vi^	2.896 (4)	C19···O3^vi^	2.940 (4)
O5···C5^vii^	3.362 (5)	C20···O3^vi^	3.305 (5)
O5···N2	3.035 (5)	C22···O1	3.293 (5)
O5···C3^viii^	3.372 (5)	C22···O6^vi^	3.324 (5)
O5···C7^vii^	3.266 (5)	C23···O1	3.358 (6)
O5···O1^vii^	3.226 (4)	C23···O4^v^	3.342 (6)
O5···C6^vii^	3.393 (5)	C22···H5	3.0600
O6···C22^v^	3.324 (5)	H2···O2	2.8100
O6···C10^ix^	3.266 (5)	H2···N2^x^	2.8800
O6···N2	3.091 (5)	H3···O5^x^	2.5900
O1···H9	2.5100	H4···O3^vi^	2.5700
O1···H11^ii^	2.7200	H5···C22	3.0600
O1···H5	2.4800	H5···O1	2.4800
O1···H22	2.9200	H9···O1	2.5100
O2···H2	2.8100	H9···N1^ii^	2.8300
O2···H23^x^	2.7500	H10···O6^xiii^	2.7200
O2···H15^iii^	2.5800	H10···O3^ii^	2.6600
O3···H4^v^	2.5700	H11···O5^iii^	2.7300
O3···H10^iv^	2.6600	H11···O1^iv^	2.7200
O4···H21	2.5000	H14···O5	2.8200
O4···H17	2.4600	H14···N1^xi^	2.8900
O4···H23^vi^	2.6800	H15···O2^xi^	2.5800
O5···H11^xi^	2.7300	H16···O6^xiv^	2.6800
O5···H14	2.8200	H17···O4	2.4600
O5···H3^viii^	2.5900	H21···O4	2.5000
O6···H16^xii^	2.6800	H21···N2^vi^	2.8500
O6···H10^ix^	2.7200	H22···O1	2.9200
O6···H22^v^	2.6000	H22···O6^vi^	2.6000
N1···O3	3.107 (5)	H23···O2^viii^	2.7500
N1···O2	3.002 (5)	H23···O4^v^	2.6800
			
O2—S1—O3	117.15 (15)	C6—C5—H5	119.00
O2—S1—C1	108.87 (15)	C8—C9—H9	120.00
O2—S1—C12	108.99 (18)	C10—C9—H9	120.00
O3—S1—C1	108.40 (14)	C9—C10—H10	120.00
O3—S1—C12	108.26 (18)	C11—C10—H10	120.00
C1—S1—C12	104.43 (15)	N1—C11—H11	119.00
O5—S2—C13	108.38 (18)	C10—C11—H11	119.00
O5—S2—C24	109.37 (18)	S2—C13—C14	115.1 (2)
O6—S2—C13	108.47 (17)	S2—C13—C18	123.6 (3)
O6—S2—C24	107.31 (18)	C14—C13—C18	121.2 (3)
C13—S2—C24	104.53 (16)	C13—C14—C15	118.9 (3)
O5—S2—O6	117.97 (16)	C14—C15—C16	121.2 (4)
C11—N1—C12	116.4 (4)	C15—C16—C17	120.2 (4)
C23—N2—C24	116.8 (4)	C16—C17—C18	120.7 (4)
S1—C1—C2	115.0 (2)	C13—C18—C17	117.8 (3)
S1—C1—C6	123.2 (3)	C13—C18—C19	123.9 (3)
C2—C1—C6	121.8 (3)	C17—C18—C19	118.3 (3)
C1—C2—C3	119.1 (3)	O4—C19—C18	120.2 (3)
C2—C3—C4	120.6 (4)	O4—C19—C20	119.8 (3)
C3—C4—C5	119.7 (4)	C18—C19—C20	120.0 (3)
C4—C5—C6	121.5 (3)	C19—C20—C21	119.5 (3)
C1—C6—C5	117.3 (3)	C19—C20—C24	123.8 (3)
C5—C6—C7	118.9 (3)	C21—C20—C24	116.7 (3)
C1—C6—C7	123.8 (3)	C20—C21—C22	119.8 (4)
O1—C7—C8	119.9 (3)	C21—C22—C23	119.6 (4)
C6—C7—C8	120.1 (3)	N2—C23—C22	122.6 (4)
O1—C7—C6	120.1 (3)	S2—C24—N2	112.0 (3)
C7—C8—C9	119.5 (3)	S2—C24—C20	123.4 (3)
C7—C8—C12	125.0 (3)	N2—C24—C20	124.5 (3)
C9—C8—C12	115.5 (3)	C13—C14—H14	121.00
C8—C9—C10	119.6 (4)	C15—C14—H14	120.00
C9—C10—C11	119.7 (4)	C14—C15—H15	119.00
N1—C11—C10	122.5 (4)	C16—C15—H15	119.00
S1—C12—N1	111.3 (3)	C15—C16—H16	120.00
S1—C12—C8	122.5 (3)	C17—C16—H16	120.00
N1—C12—C8	126.3 (3)	C16—C17—H17	120.00
C3—C2—H2	120.00	C18—C17—H17	120.00
C1—C2—H2	120.00	C20—C21—H21	120.00
C2—C3—H3	120.00	C22—C21—H21	120.00
C4—C3—H3	120.00	C21—C22—H22	120.00
C5—C4—H4	120.00	C23—C22—H22	120.00
C3—C4—H4	120.00	N2—C23—H23	119.00
C4—C5—H5	119.00	C22—C23—H23	119.00
			
O2—S1—C1—C2	−56.2 (3)	C1—C6—C7—C8	−6.4 (5)
O2—S1—C1—C6	125.4 (3)	C5—C6—C7—O1	−6.9 (5)
O3—S1—C1—C2	72.3 (3)	C5—C6—C7—C8	173.7 (3)
O3—S1—C1—C6	−106.1 (3)	O1—C7—C8—C9	7.0 (6)
C12—S1—C1—C2	−172.5 (3)	O1—C7—C8—C12	−171.8 (4)
C12—S1—C1—C6	9.1 (3)	C6—C7—C8—C9	−173.6 (4)
O2—S1—C12—N1	57.7 (4)	C6—C7—C8—C12	7.6 (6)
O2—S1—C12—C8	−124.3 (4)	C7—C8—C9—C10	−177.9 (4)
O3—S1—C12—N1	−70.7 (4)	C12—C8—C9—C10	1.0 (7)
O3—S1—C12—C8	107.3 (4)	C7—C8—C12—S1	0.6 (6)
C1—S1—C12—N1	173.9 (4)	C7—C8—C12—N1	178.3 (5)
C1—S1—C12—C8	−8.0 (4)	C9—C8—C12—S1	−178.3 (4)
O5—S2—C13—C18	−125.2 (3)	C9—C8—C12—N1	−0.5 (7)
O6—S2—C13—C14	−72.0 (4)	C8—C9—C10—C11	−0.2 (8)
O6—S2—C13—C18	105.6 (3)	C9—C10—C11—N1	−1.4 (8)
C24—S2—C13—C14	173.7 (3)	S2—C13—C14—C15	176.3 (4)
C24—S2—C13—C18	−8.7 (4)	C18—C13—C14—C15	−1.4 (7)
O5—S2—C24—N2	−58.7 (4)	S2—C13—C18—C17	−176.1 (3)
O5—S2—C24—C20	124.3 (4)	S2—C13—C18—C19	3.9 (5)
O6—S2—C24—N2	70.3 (4)	C14—C13—C18—C17	1.3 (6)
O6—S2—C24—C20	−106.7 (4)	C14—C13—C18—C19	−178.7 (4)
C13—S2—C24—N2	−174.6 (3)	C13—C14—C15—C16	0.8 (8)
C13—S2—C24—C20	8.4 (4)	C14—C15—C16—C17	−0.1 (9)
O5—S2—C13—C14	57.2 (4)	C15—C16—C17—C18	0.0 (8)
C11—N1—C12—C8	−0.9 (8)	C16—C17—C18—C13	−0.6 (6)
C12—N1—C11—C10	1.8 (8)	C16—C17—C18—C19	179.4 (4)
C11—N1—C12—S1	177.1 (4)	C13—C18—C19—O4	−178.2 (3)
C23—N2—C24—S2	−176.8 (3)	C13—C18—C19—C20	3.2 (5)
C23—N2—C24—C20	0.1 (7)	C17—C18—C19—O4	1.8 (5)
C24—N2—C23—C22	−0.3 (7)	C17—C18—C19—C20	−176.8 (3)
S1—C1—C6—C5	177.0 (3)	O4—C19—C20—C21	−2.2 (6)
S1—C1—C2—C3	−176.7 (3)	O4—C19—C20—C24	177.9 (4)
C6—C1—C2—C3	1.7 (6)	C18—C19—C20—C21	176.4 (4)
C2—C1—C6—C7	178.8 (3)	C18—C19—C20—C24	−3.5 (6)
S1—C1—C6—C7	−2.9 (4)	C19—C20—C21—C22	179.7 (4)
C2—C1—C6—C5	−1.3 (5)	C24—C20—C21—C22	−0.4 (7)
C1—C2—C3—C4	−0.4 (7)	C19—C20—C24—S2	−3.3 (6)
C2—C3—C4—C5	−1.2 (7)	C19—C20—C24—N2	−179.9 (4)
C3—C4—C5—C6	1.7 (7)	C21—C20—C24—S2	176.8 (4)
C4—C5—C6—C7	179.5 (4)	C21—C20—C24—N2	0.2 (7)
C4—C5—C6—C1	−0.4 (6)	C20—C21—C22—C23	0.3 (8)
C1—C6—C7—O1	173.0 (3)	C21—C22—C23—N2	0.1 (7)

Symmetry codes: (i) −*x*+1, −*y*+1, *z*−1/2; (ii) *x*−1/2, −*y*, *z*; (iii) −*x*+3/2, *y*−1, *z*−1/2; (iv) *x*+1/2, −*y*, *z*; (v) *x*+1/2, −*y*+1, *z*; (vi) *x*−1/2, −*y*+1, *z*; (vii) −*x*+1, −*y*+1, *z*+1/2; (viii) −*x*+3/2, *y*, *z*+1/2; (ix) *x*, *y*+1, *z*; (x) −*x*+3/2, *y*, *z*−1/2; (xi) −*x*+3/2, *y*+1, *z*+1/2; (xii) *x*+1/2, −*y*+2, *z*; (xiii) *x*, *y*−1, *z*; (xiv) *x*−1/2, −*y*+2, *z*.

### Hydrogen-bond geometry (Å, °)

**Table:**

Cg1 and Cg2 are the centroids of the S1/C1/C6/C7/C8/C12 and S2/C13/C18/C19/C20/C24 rings, respectively.

**Table:**

*D*—H···*A*	*D*—H	H···*A*	*D*···*A*	*D*—H···*A*
C3—H3···O5^x^	0.93	2.59	3.372 (5)	142
C4—H4···O3^vi^	0.93	2.57	3.472 (5)	164
C15—H15···O2^xi^	0.93	2.58	3.367 (5)	143
S1—O3···Cg2^xi^	1.434 (3)	3.212 (4)	4.421 (3)	141.14 (14)
C19—O4···Cg1^vi^	1.207 (4)	2.869 (3)	3.585 (4)	117.2 (2)

Symmetry codes: (x) −*x*+3/2, *y*, *z*−1/2; (vi) *x*−1/2, −*y*+1, *z*; (xi) −*x*+3/2, *y*+1, *z*+1/2.
